# Gait and Cognition in Parkinson’s Disease: Cognitive Impairment Is Inadequately Reflected by Gait Performance during Dual Task

**DOI:** 10.3389/fneur.2017.00550

**Published:** 2017-10-26

**Authors:** Heiko Gaßner, Franz Marxreiter, Simon Steib, Zacharias Kohl, Johannes C. M. Schlachetzki, Werner Adler, Bjoern M. Eskofier, Klaus Pfeifer, Jürgen Winkler, Jochen Klucken

**Affiliations:** ^1^Department of Molecular Neurology, University Hospital Erlangen, Friedrich-Alexander University Erlangen-Nürnberg (FAU), Erlangen, Germany; ^2^Institute for Sport Science and Sport, Friedrich-Alexander University Erlangen-Nürnberg (FAU), Erlangen, Germany; ^3^Department of Medical Informatics, Biometry and Epidemiology, Friedrich-Alexander University Erlangen-Nürnberg (FAU), Erlangen, Germany; ^4^Chair for Machine Learning and Data Analytics, Friedrich-Alexander University Erlangen-Nürnberg (FAU), Erlangen, Germany

**Keywords:** Parkinson’s disease, gait, cognition, dual task, gait analysis, Montreal Cognitive Assessment

## Abstract

**Introduction:**

Cognitive and gait deficits are common symptoms in Parkinson’s disease (PD). Motor-cognitive dual tasks (DTs) are used to explore the interplay between gait and cognition. However, it is unclear if DT gait performance is indicative for cognitive impairment. Therefore, the aim of this study was to investigate if cognitive deficits are reflected by DT costs of spatiotemporal gait parameters.

**Methods:**

Cognitive function, single task (ST) and DT gait performance were investigated in 67 PD patients. Cognition was assessed by the Montreal Cognitive Assessment (MoCA) followed by a standardized, sensor-based gait test and the identical gait test while subtracting serial 3’s. Cognitive impairment was defined by a MoCA score <26. DT costs in gait parameters [(DT − ST)/ST × 100] were calculated as a measure of DT effect on gait. Correlation analysis was used to evaluate the association between MoCA performance and gait parameters. In a linear regression model, DT gait costs and clinical confounders (age, gender, disease duration, motor impairment, medication, and depression) were correlated to cognitive performance. In a subgroup analysis, we compared matched groups of cognitively impaired and unimpaired PD patients regarding differences in ST, DT, and DT gait costs.

**Results:**

Correlation analysis revealed weak correlations between MoCA score and DT costs of gait parameters (*r*/*r*_Sp_ ≤ 0.3). DT costs of stride length, swing time variability, and maximum toe clearance (|*r*/*r*_Sp_| > 0.2) were included in a regression analysis. The parameters only explain 8% of the cognitive variance. In combination with clinical confounders, regression analysis showed that these gait parameters explained 30% of MoCA performance. Group comparison revealed strong DT effects within both groups (large effect sizes), but significant between-group effects in DT gait costs were not observed.

**Conclusion:**

These findings suggest that DT gait performance is not indicative for cognitive impairment in PD. DT effects on gait parameters were substantial in cognitively impaired and unimpaired patients, thereby potentially overlaying the effect of cognitive impairment on DT gait costs. Limits of the MoCA in detecting motor-function specific cognitive performance or variable individual response to the DT as influencing factors cannot be excluded. Therefore, DT gait parameters as marker for cognitive performance should be carefully interpreted in the clinical context.

## Introduction

Cognitive and gait deficits are common symptoms in Parkinson’s disease (PD) that worsen with disease progression (1). Motor-cognitive dual tasks (DTs) (e.g., walking while talking) as a method to investigate the interplay between gait and cognition require both motor control and cognition to perform the DT (2, 3). It is well known that DTs affect gait even in healthy individuals by reducing gait velocity (4, 5). The interference between motor control and cognitive demand is important for motor-impaired patients in everyday life activities. In mild to moderately affected PD patients, cognitive DTs during walking (subtracting numbers, digit span DT) reduced gait performance and increased gait variability (4, 6–8). This indicates that dual tasking may be responsible for trip and fall risk in patients with motor impairments due to additionally challenges on motor control (9–11).

Walking is a complex task in which cognitive resources continuously monitor bilateral coordination and dynamic postural control, both necessary for the walking process including cognitive-motor control (3). In this context, a systematic review revealed that there is a direct relationship between cognitive deficit severity and gait abnormalities in patients with dementia (12). Cognitive impairment in PD typically consists of deficits in attention, executive and visuospatial functions, as well as memory resources (13). Even in early PD, attention and executive function deficits are features of basal ganglia pathology (14) and necessary to appropriately allocate cognitive resources for the optimal performance of simultaneous tasks (15). Therefore, it is not surprising that attention and executive function are associated with DT performance (4, 16, 17).

In clinical routine the Montreal Cognitive Assessment (MoCA) is a widely used and international accepted scale to evaluate the cognitive status in PD (18–22). It is a 30-point scale that evaluates visuospatial function, language, digit span, executive functions, attention, and memory (23). It has been previously shown that executive functions, attention, visuospatial abilities, and memory are associated with gait impairment in PD (3, 5, 15, 24, 25). In particular, the association between executive functions and DT gait performance has been described (4, 6, 15). Evaluating DT situations in PD is useful since cognitive functions and motor control—both often impaired in PD—are examined at the same time. DT costs are a common measure to evaluate the effect of the secondary (cognitive) task on the primary task (walking) and have been demonstrated to reflect impaired DT gait performance in PD (5).

With regard to the interplay between gait, cognition, and dual tasking, the majority of studies assessed the effect of DT on gait or the effect of cognitive deficits on gait (5, 6, 16, 25). However, it is not precisely characterized whether cognitive performance can be predicted by gait performance under DT condition. One study observed that DT affects swing time, step length variability, and single/double support time ratio more pronounced in PD patients with mild cognitive impairment compared with controls (24). This indicates that gait might serve as an objective clinical biomarker for cognitive gait control which could support fall risk detection and fall prevention in PD. Since cognitive deficits are correlated with a larger fall risk (26) and associated with a shorter survival time (27, 28) it is worth studying if cognitive deficits may be mirrored by gait performance to support the clinical routine with quantitative data and early detect risk factors for falls.

The aim of this study was to investigate if cognitive performance evaluated with a clinical cognitive assessment (MoCA) may be reflected by DT costs of spatiotemporal gait parameters. In this context it is interesting to detect which DT gait parameters are sensitive to cognitive impairment.

We hypothesize that cognitive deficits correlate with DT gait performance in PD patients and that cognitive deficits can be predicted by DT gait parameters. To test this hypothesis, three analyses were performed: (1) correlation analysis between MoCA score and DT costs for each gait parameter separately [DT costs are defined as the percentage change between single task (ST) and DT gait parameters], (2) linear regression analysis that includes most relevant gait parameters (based on the correlations) and confounders of DT gait performance (e.g., age and disease duration), and (3) a comparison of cognitively impaired and unimpaired PD patients in two groups that are matched by clinical confounders. In summary, we observed subtle correlations between cognitive performance and DT gait costs. Cognitively impaired and unimpaired PD patients were equally challenged by the DT gait test.

## Materials and Methods

### Participants

Parkinson’s disease patients were selected from a large stratified patient cohort (*n* = 406) visiting the Movement Disorders Outpatient Clinic of the Department of Molecular Neurology at the University Hospital Erlangen, Germany between July 2014 and March 2016. Sporadic PD was defined according to the Guidelines of the German Association for Neurology (DGN), which are similar to the UK PD Society Brain Bank criteria (29). PD patients with Hoehn and Yahr disease stage (H&Y) between I and III were included, they were able to walk independently without a walking aid, and underwent a cognitive assessment using MoCA. Patients were not selected if they also showed troublesome or disabling motor fluctuations (“freezing of gait,” “sudden-offs,” “end-of-dose wearing offs,” “peak dose dyskinesia”) (30, 31) or non-PD related causes of gait impairments (e.g. due to spinal or orthopedic surgery), atypical Parkinson syndromes, spasticity, stroke, neuropathy, myelopathy, and hydrocephalus. PD patients were clinically assessed by a movement disorders specialist using the Unified Parkinson Disease Rating Scale part III (UPDRS-III) (32). All PD patients were clinically (UPDRS-III and MoCA) (23) and biomechanically (gait analysis) (33, 34) investigated in stable ON medication without presence of clinically relevant motor fluctuations during the assessments. Stable ON medication indicated that patients were on medication, received best medical treatment, and took their medication as suggested.

**Figure 1:**
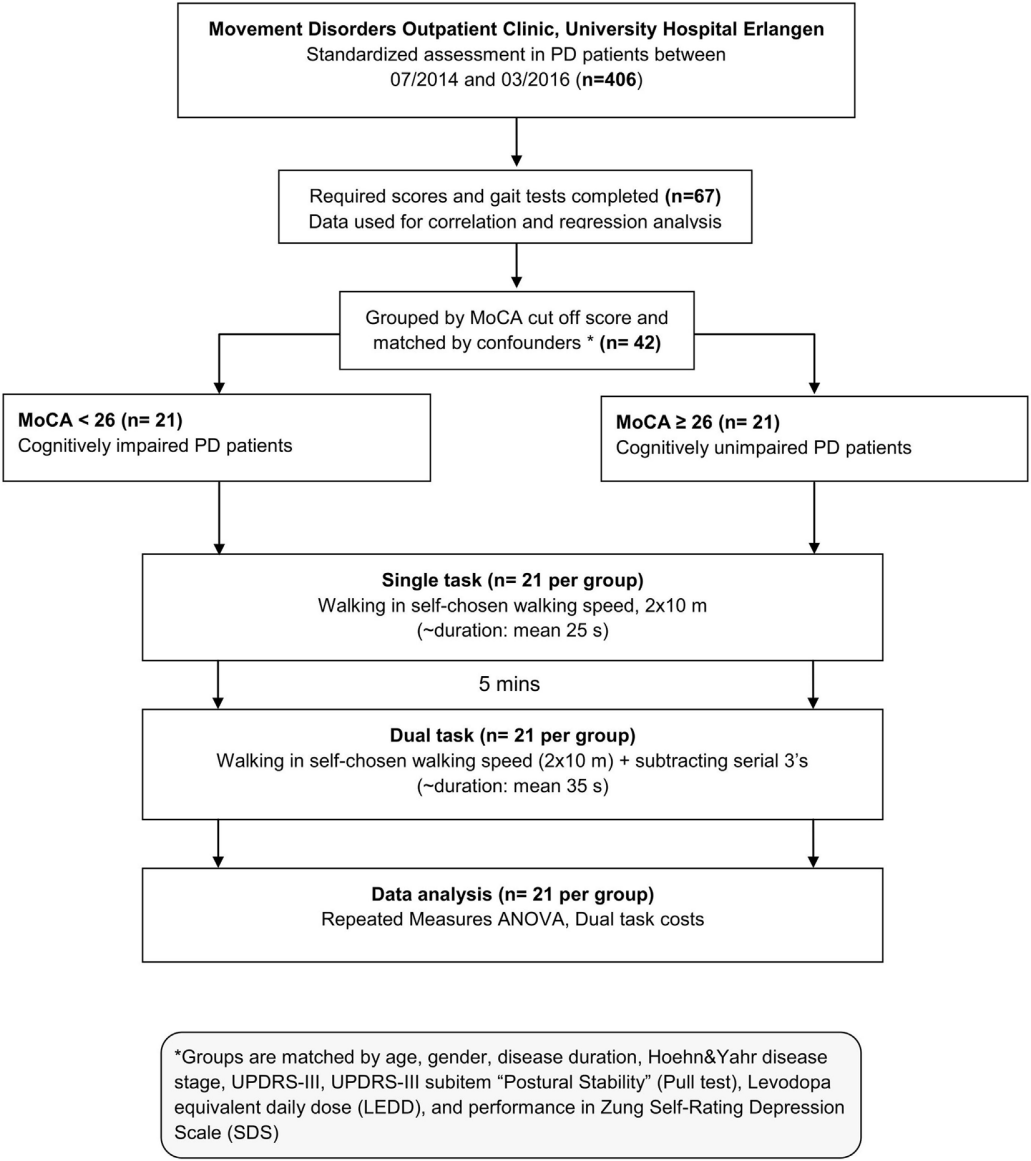
Flowchart.

From 406 PD patients, 67 patients completed all required scores and gait tests in this retrospective study design. In a first step, this data set was used for correlations and linear regression analysis investigating the association between cognitive and DT gait performance. Secondly, for comparison between cognitively impaired and unimpaired PD patients, we stratified participants by MoCA cutoff score (<26; ≥26) (35) and pairwise matched PD patients by age, gender, disease duration, H&Y, UPDRS-III, UPDRS-III subitem “Postural Stability” (Pull test), levodopa equivalent daily dose (LEDD) (36), and performance in Zung Self-Rating Depression Scale (SDS) (37), all of them been detected as confounders to DT performance (17, 24). PD patients included in this study were pairwise matched resulting in 21 patients in each group. Matched groups only differ in MoCA score to separately evaluate cognitive influence on gait (see flowchart, Figure 1). Participants in both groups did not differ either in the Postural Instability and Gait Difficulty (PIGD) subscore of the UPDRS-III (38), nor in the PIGD/UPDRS-III ratio meaning that gait and balance impairment (PIGD) relative to the global motor impairment (UPDRS-III) are similar in both groups. Characteristics of the matched study population were presented in Table 1. The study was approved by the local ethics committee (IRB-approval-Re. No. 4208, IRB, Medical Faculty, Friedrich-Alexander University Erlangen-Nürnberg, Germany), and all participants gave written informed consent according to the Declaration of Helsinki. Despite some patients had a MoCA score of 17 [<21 relating to PD dementia (35)], however, this level of cognitive performance was sufficient for their everyday activities and these patients did not have a legal guardian. The ability to give fully informed consent was decided during the routine clinical examination.

**Table 1:** Characteristics of study population. Mann–Whitney *U*-test was used to evaluate baseline differences between groups.

	MOCA < 26 (*n* = 21)	MOCA ≥ 26 (*n* = 21)	*p*-Value
Age (years, mean ± SD) (range)	65.1 ± 10.6 (46–82)	64.2 ± 8.0 (45–75)	0.756^a^
Gender (male:female)	17:4	16:5	0.707^b^
Education (years, mean ± SD)	13.4 ± 2.4	14.6 ± 2.7	0.152
Disease duration (years, mean ± SD) (range)	6.2 ± 3.5 (2–16)	7.1 ± 2.6 (3–13)	0.199
H&Y (mean ± SD) (range)	2.5 ± 0.8 (1–3.5)	2.4 ± 0.7 (1–3.5)	0.603
UPDRS motor score (mean ± SD) (range)	17.2 ± 8.6 (5–36)	18.8 ± 7.9 (7–39)	0.442
Pull test score (mean ± SD) (range)	0.8 ± 0.5 (0–2)	0.7 ± 0.6 (0–2)	0.542
PIGD (mean ± SD) (range)	2.9 ± 1.9 (0–6)	2.6 ± 1.4 (0–5)	0.591
PIGD/UPDRS-III ratio (%, mean ± SD) (range)	16.7 ± 12.2 (0–40)	15.0 ± 8.6 (0–33)	0.890
LEDD (mg/day, mean ± SD) (range)	624.4 ± 270.4 (257–1391)	649.2 ± 297.0 (180–1141)	0.778^a^
Zung Depression Scale	45.3 ± 11.2	44.2 ± 9.7	0.742^a^
MoCA (mean ± SD) (range)	23.1 ± 2.5 (17–25)	28.1 ± 1.4 (26–30)	**0.000**
Visuospatial/executive	3.8 ± 1.4	4.8 ± 0.5	**0.007**
Naming	3.0 ± 0.2	3.0 ± 0.0	0.317
Attention	5.1 ± 0.9	5.8 ± 0.5	**0.005**
Language	1.6 ± 0.9	2.5 ± 0.5	**0.001**
Abstraction	1.5 ± 0.5	1.9 ± 0.4	**0.010**
Memory—delayed recall	2.3 ± 1.5	4.2 ± 1.0	**0.000**
Orientation	5.8 ± 0.5	6.0 ± 0.2	0.152

*H&Y, Hoehn and Yahr disease stage; PIGD, Postural Instability and Gait Difficulty score; LEDD, levodopa equivalent daily dose; MoCA, Montreal Cognitive Assessment; UPDRS-III, Unified Parkinson Disease Rating Scale part III*.

*Groups are matched by age, gender, and clinical scores and only differ concerning MoCA score. Bold numbers indicate significance*.

*^a^One-way ANOVA*.

*^b^χ^2^ test*.

### Cognitive Assessment and Sensor-Based Gait Analysis

The MoCA (23) was performed and evaluated by a trained instructor. PD patients were stratified according to the established MoCA cutoff score <26 (cognitively impaired) and ≥26 (cognitively unimpaired) (35) and matched by clinical confounders mentioned above. Gait analysis was performed on a predefined 2 m × 10 m walking distance at self-chosen and comfortable speed (ST) including a turn (180°) after the first 10 m. Following the ST, PD patients repeated the test and were challenged to count backwards in steps of three starting from a number >100 while performing the 2 m × 10 m walk (DT) (6, 39) (see Figure 1). Participants were not instructed to prioritize walking or counting. DT costs for gait parameters as a measure of the effect of the cognitive task on gait were calculated by the following formula (2):

$$\text{Dual task costs}=\frac{\left(\text{Dual task gait parameter}-\text{Single task gait parameter}\right)}{\text{Single task gait parameter}}\times 100.$$

Gait analysis was performed using an instrumented gait analysis system which consists of inertial sensors (gyroscopes and accelerometers, SHIMMER 2 sensors, Shimmer Research Ltd., Dublin, Ireland). The biosensors were laterally attached to the heel part of both shoes and provided kinematic gait parameters. Recordings were performed using an accelerometer range of ±6 g (sensitivity 300 mV/g), a gyroscope range of ±500°/s (sensitivity 2 mV/°/s), and a sampling rate of 102.4 Hz. Sensor signals were transmitted via Bluetooth^®^ to a tablet computer and stored for subsequent data analysis (40). Inertial sensor data were processed with a pattern recognition algorithm for computing clinically relevant spatiotemporal gait parameters (stride length, gait velocity, cadence, stride time, stance time, swing time, heel strike (HS)/toe off (TO) angle, and maximum foot clearance) (33). HS angle is defined as the angle between foot and the floor at initial foot contact (beginning of the stance phase). TO angle is defined as the angle between foot and the ground during push-off at the end of the stance phase (41, 42). Coefficient of variance (CV) = SD/mean was calculated for stride time, swing time, stance time, and stride length. Only straight strides were automatically detected and used for gait parameter calculations as described, turning steps were automatically excluded (34). Gait velocity, stride length, cadence, stride time, and maximum toe clearance (max TC) were normalized to the height of the participants to control for body height differences.

### Statistical Analysis

Correlation analysis between MoCA score and DT gait costs was performed using Pearson’s correlation (*r*) or Spearman’s rank correlation (*r*_Sp_) in cases of not normally distributed variables (stance time, swing time, cadence, and CV of stride time, stance time, swing time, stride length). In a regression analysis, DT costs of most relevant gait parameters (|*r*/*r*_Sp|_ > 0.2) were combined and correlated to cognitive performance. In a second regression model, we additionally included clinical confounders that may interact with DT gait performance to assess the combined effect of DT gait costs and confounders on cognition. Furthermore, group comparison of matched PD patients was assessed to evaluate effects of DT and interaction effects between cognitively unimpaired and impaired PD patients. Mann–Whitney *U*-test was used to evaluate baseline differences in non-parametric or not normally distributed confounders including disease duration, and clinical scores between matched groups of cognitively impaired and unimpaired PD patients. Differences in normally distributed parameters with homogeneous variances (age, LEDD, and Zung Depression scale) were compared between groups using one-way ANOVA. Normality of data was tested by Shapiro–Wilk test and variance homogeneity by Levene test. A repeated measures ANOVA was used to evaluate the effect of the motor-cognitive DT on spatiotemporal gait parameters. Main effects between ST and DT condition (task) were analyzed for the entire cohort and for each group separately (within-group effect). Non-parametric measures were used for parameters that are not normally distributed (paired Wilcoxon test for within-group effects and Mann–Whitney *U*-test for between-group effects). DT costs were calculated to determine the effect of the secondary cognitive task (DT) on the primary task (gait parameters) (2). One-way ANOVA was used to detect differences of DT gait costs between cognitively impaired and unimpaired PD patients. In case of not normally distributed parameters Mann–Whitney *U*-test was used. Eta squared (η^2^) given by ANOVA and calculated from *Z* values given by Wilcoxon or Mann–Whitney *U*-test is presented as measure of effect size. For comparison of baseline characteristics the significance level was set at α = 0.05. In all analyses including gait parameters significance level was adapted by Benjamini–Hochberg multiple testing correction (α_C_). This procedure consists of sorting the *p*-values in ascending order, and then dividing each *p*-value by its percentile rank to receive an estimated false discovery rate (43). Since in this study 13 gait parameters were compared with MoCA score, adjusted significance levels are α_C_ = 0.004 for the lowest *p*-value, α_C_ = 0.008 for the next highest *p*-value, and so on. All statistical analyses were performed using SPSS software package version 21 (IBM Corp. Released 2012. IBM SPSS Statistics for Windows, Version 21.0. Armonk, NY, USA: IBM Corp.).

## Results

### Correlation Analysis between MoCA and DT Costs

**Figure 2:**
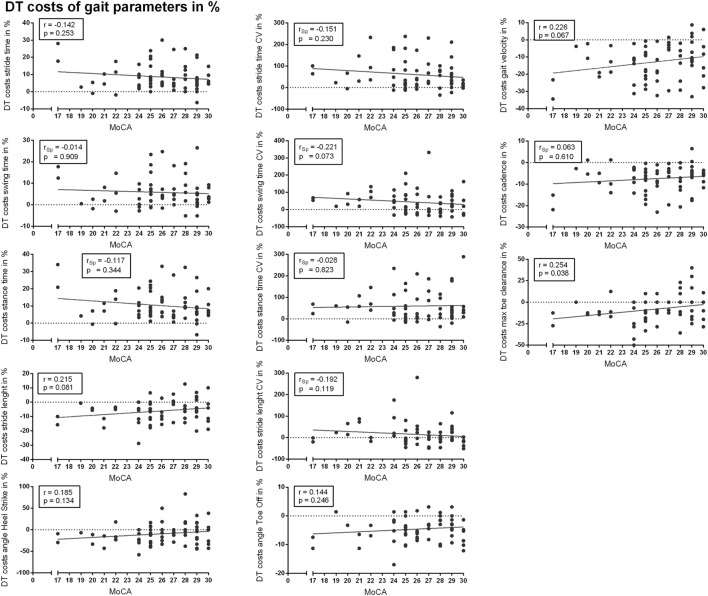
Correlation analysis between cognitive performance [Montreal Cognitive Assessment (MoCA)] and dual task (DT) costs of spatiotemporal gait parameters. *r*, Pearson’s correlation coefficient; *r*_Sp_, Spearman’s rank correlation coefficient.

Correlation analysis revealed subtle correlations between MoCA score and DT costs of spatiotemporal gait parameters (*r*/*r*_Sp_ ≤ 0.3). Most relevant but not significant correlations were detected for DT costs in max TC (*r* = 0.254, *p* = 0.038, α_C_ = 0.004), gait velocity (*r* = 0.226, *p* = 0.067, α_C_ = 0.008), swing time variability (*r*_Sp_ = −0.221, *p* = 0.073, α_C_ = 0.012), and stride length (*r* = 0.215, *p* = 0.081, α_C_ = 0.015). All correlations are presented in Figure 2. Subtle correlations were observed between MoCA and gait parameters in ST and DT condition (Tables S1 and S2 in Supplementary Material).

### Regression Analysis

Dual task costs of gait parameters (stride length, swing time variability, and max TC) that showed correlation coefficients |*r*/*r*_Sp_| > 0.2 were included in a linear regression analysis. Gait velocity was excluded in this regression model since it shows strong collinearity with stride length (*r* > 0.7). Linear regression analysis revealed that DT costs of stride length, swing time variability, and max TC only explain 8.6% of the variance in cognitive performance (MoCA) based on the given *R*^2^ value. By additionally including clinical confounders in the present regression model, 29.4% of MoCA performance is explained.

### MoCA Performance in Matched PD Groups

Using the MoCA, PD patients (*n* = 42) reached a mean sum score of 25.4 ± 3.3 with a range from 17 to 30 points. The PD cohort was stratified into two groups (*n* = 21 each) by the established MoCA cutoff score of 26 and matched by the clinical confounders age, gender, disease duration, H&Y, UPDRS-III, Pull test, LEDD, and SDS performance. Cognitively impaired PD patients reached significantly lower subscores in the MoCA subcategories “visuospatial/executive” (*p* = 0.007; η^2^ = 0.176), “attention” (*p* = 0.005; η^2^ = 0.190), “language” (*p* = 0.001; η^2^ = 261), “abstraction” (*p* = 0.010; η^2^ = 0.159), and “memory” (*p* = 0.000; η^2^ = 0.347), whereas no difference was observed for categories “naming” (*p* = 0.317; η^2^ = 0.024), and “orientation” (*p* = 0.152; η^2^ = 0.049) between groups.

#### Gait Parameters in Single and DT Condition

Sensor-based gait analysis revealed differences in spatiotemporal gait parameters between groups in the ST walking condition. Cognitively impaired PD patients showed reduced stride length and gait velocity compared with PD patients without cognitive impairment but without reaching significance level. TO angle was significantly reduced in the cognitively impaired group (Table 2). During DT walking, all gait parameters were comparable between groups and did not show significant differences. Repeated measures ANOVA and paired Wilcoxon test revealed significant effects of the DT in all spatiotemporal gait parameters with exception of stride length CV. Strong effect sizes in particular for gait velocity (η^2^ = 0.769), stride length (η^2^ = 0.603), cadence (η^2^ = 0.759), and TO angle (η^2^ = 0.777) indicate that these parameters substantially reflect gait impairment under DT (Table 3). Stratified by the MoCA cutoff, gait was similarly significantly impaired in both groups under DT condition. In the cognitively impaired group all gait parameters except of stride length CV were affected by DT, in the cognitively unimpaired group all gait parameters except of stride length CV and HS angle significantly deteriorated by the DT condition (Table 4).

**Table 2:** One-way ANOVA and Mann–Whitney *U*-test were performed to compare spatiotemporal gait parameters in single task (ST) (walking) between cognitively impaired (MoCA < 26) and unimpaired (MoCA ≥ 26) Parkinson’s disease patients (A); gait parameters of dual task (DT) (walking + counting backwards) are compared between groups in (B).

Gait parameter	Cognitively impaired		Cognitively unimpaired		*F* (1,40); *Z* value^a^	*p*	η^2^	α_C_
	Mean	SD	Mean	SD				
**(A) Single task**								

Stride time normalized (s)	1.04	0.07	1.04	0.08	0.004	0.951	0.000	0.046
Stride time CV (%)	2.39	0.5	2.34	0.57	−0.516^a^	0.606	0.006	0.035
Swing time (%)	35.7	1.19	35.8	1.55	−0.176^a^	0.860	0.001	0.042
Swing time CV (%)	3.21	1.07	2.84	0.50	−0.743^a^	0.458	0.013	0.031
Stance time (%)	64.3	1.19	64.2	1.55	−0.176^a^	0.860	0.001	0.038
Stance time CV (%)	3.05	0.66	3.12	0.85	−0.038^a^	0.970	0.000	0.050
Stride length normalized (m)	1.36	0.15	1.49	0.15	6.700	0.013	0.143	0.008
Stride length CV (%)	6.52	2.04	6.07	1.67	−0.856^a^	0.392	0.017	0.027
HS angle (°)	11.0	4.17	12.93	6.34	1.355	0.251	0.033	0.019
TO angle (°)	−63.10	7.02	−69.69	6.36	**10.189**	**0.003**	**0.203**	0.004
max TC normalized (cm)	8.46	2.16	9.51	3.30	1.486	0.230	0.036	0.015
Cadence norm. (strides/min)	57.23	4.07	58.58	6.35	−1.094^a^	0.274	0.028	0.023
Gait velocity normalized (m/s)	1.31	0.19	1.44	0.19	5.370	0.026	0.118	0.012

**(B) Dual task**								

Stride time normalized (s)	1.31	0.19	1.44	0.20	5.177	0.028	0.115	0.008
Stride time CV (%)	3.76	2.24	4.45	3.03	−0.566^a^	0.571	0.008	0.031
Swing time (%)	34.9	1.35	34.8	2.22	−0.456^a^	0.642	0.005	0.038
Swing time CV (%)	4.57	1.75	4.98	3.18	−0.528^a^	0.597	0.007	0.035
Stance time (%)	65.1	1.35	65.2	2.22	−0.428^a^	0.669	0.004	0.042
Stance time CV (%)	4.63	3.07	5.5	3.33	−0.919^a^	0.358	0.020	0.023
Stride length normalized (m)	1.26	0.15	1.38	0.20	4.890	0.033	0.109	0.012
Stride length CV (%)	7.09	3.44	7.53	4.29	−0.365^a^	0.715	0.003	0.050
HS angle (°)	9.71	4.27	12.5	6.82	2.561	0.117	0.060	0.019
TO angle (°)	−59.6	7.46	−65.2	6.22	6.974	0.012	0.148	0.004
max TC normalized (cm)	7.5	2.2	9.02	3.41	2.961	0.093	0.069	0.015
Cadence norm. (strides/min)	52.4	4.6	53.1	7.30	−0.717^a^	0.473	0.012	0.004
Gait velocity normalized (m/s)	0.97	0.82	0.96	0.99	0.138	0.712	0.003	0.046

*HS, heel strike; TO, toe off; max TC, maximum toe clearance; CV, coefficient of variance; SD, standard deviation*.

*Significance level was adapted by Benjamini–Hochberg multiple testing correction (α_C_). Bold numbers indicate significance*.

*^a^Mann–Whitney U-test*.

**Table 3:** Results of the repeated measures ANOVA and paired Wilcoxon test, effects of dual task for all participants (*n* = 42).

Gait parameters	Main effect task/within-group effect (*n* = 42)			
	*F* (1,40); *Z* value^a^	*p*	η^2^	α_C_
Stride time normalized (s)	**81.112**	**0.000**	**0.670**	0.004
Stride time CV (%)	**−4.614**^a^	**0.000**	**0.507**	0.008
Swing time (%)	**−4.764**^a^	**0.000**	**0.540**	0.012
Swing time CV (%)	**−3.889**^a^	**0.000**	**0.360**	0.015
Stance time (%)	**−4.770**^a^	**0.000**	**0.542**	0.019
Stance time CV (%)	**−4.192**^a^	**0.000**	**0.418**	0.023
Stride length normalized (m)	**60.703**	**0.000**	**0.603**	0.027
Stride length CV (%)	−1.338^a^	0.181	0.043	0.050
HS angle (°)	**9.226**	**0.004**	**0.187**	0.046
TO angle (°)	**139.295**	**0.000**	**0.777**	0.031
max TC normalized (cm)	**31.561**	**0.000**	**0.441**	0.035
Cadence norm. (strides/min)	**−5.645**^a^	**0.000**	**0.759**	0.038
Gait velocity normalized (m/s)	**133.402**	**0.000**	**0.769**	0.042

*HS, heel strike; TO, toe off; max TC, maximum toe clearance; CV, coefficient of variance*.

*Significance level was adapted by Benjamini–Hochberg correction for multiple comparisons (α_C_). Bold numbers indicate significance*.

*^a^Wilcoxon test*.

**Table 4:** Results of the repeated measures ANOVA and paired Wilcoxon test, within-group effects separated in cognitively impaired (*n* = 21) and unimpaired (*n* = 21) Parkinson’s disease patients.

Gait parameters	Within-group effect							
	Cognitively impaired (*n* = 21)				Cognitively unimpaired (*n* = 21)			
	*F* (1,40); *Z* value^a^	*p*	η^2^	α_C_	*F* (1,40); *Z* value^a^	*p*	η^2^	α_C_
Stride time normalized (s)	**25.674**	**0.000**	**0.562**	0.004	**58.980**	**0.000**	**0.747**	0.004
Stride time CV (%)	**−3.323**^a^	**0.001**	**0.526**	0.038	**−3.303**^a^	**0.001**	**0.520**	0.027
Swing time (%)	**−3.810**^a^	**0.000**	**0.691**	0.008	**−3.094**^a^	**0.002**	**0.456**	0.031
Swing time CV (%)	**−2.521**^a^	**0.012**	**0.303**	0.046	**−2.972**^a^	**0.003**	**0.421**	0.038
Stance time (%)	**−3.810**^a^	**0.000**	**0.691**	0.012	**−3.094**^a^	**0.002**	**0.456**	0.035
Stance time CV (%)	**−2.763**^a^	**0.006**	**0.364**	0.042	**−3.192**^a^	**0.001**	**0.485**	0.023
Stride length normalized (m)	**37.209**	**0.000**	**0.650**	0.015	**25.539**	**0.000**	**0.561**	0.008
Stride length CV (%)	−0.400^a^	0.689	0.008	0.050	−1.495^a^	0.135	0.106	0.046
HS angle (°)	**17.357**	**0.000**	**0.503**	0.019	0.718	0.407	0.035	0.050
TO angle (°)	**41.733**	**0.000**	**0.676**	0.023	**121.631**	**0.000**	**0.859**	0.012
max TC normalized (cm)	**41.846**	**0.000**	**0.677**	0.027	**5.360**	**0.031**	**0.211**	0.042
Cadence norm. (strides/min)	**−4.015**^a^	**0.000**	**0.768**	0.031	**−4.015**^a^	**0.000**	**0.768**	0.015
Gait velocity normalized (m/s)	**43.663**	**0.000**	**0.686**	0.035	**96.081**	**0.000**	**0.828**	0.019

*HS, heel strike; TO, toe off; max TC, maximum toe clearance; CV, coefficient of variance*.

*Significance level was adapted by Benjamini–Hochberg correction for multiple comparisons (α_C_). Bold numbers indicate significance*.

*^a^Wilcoxon test*.

#### DT Costs in Gait Parameters

**Figure 3:**
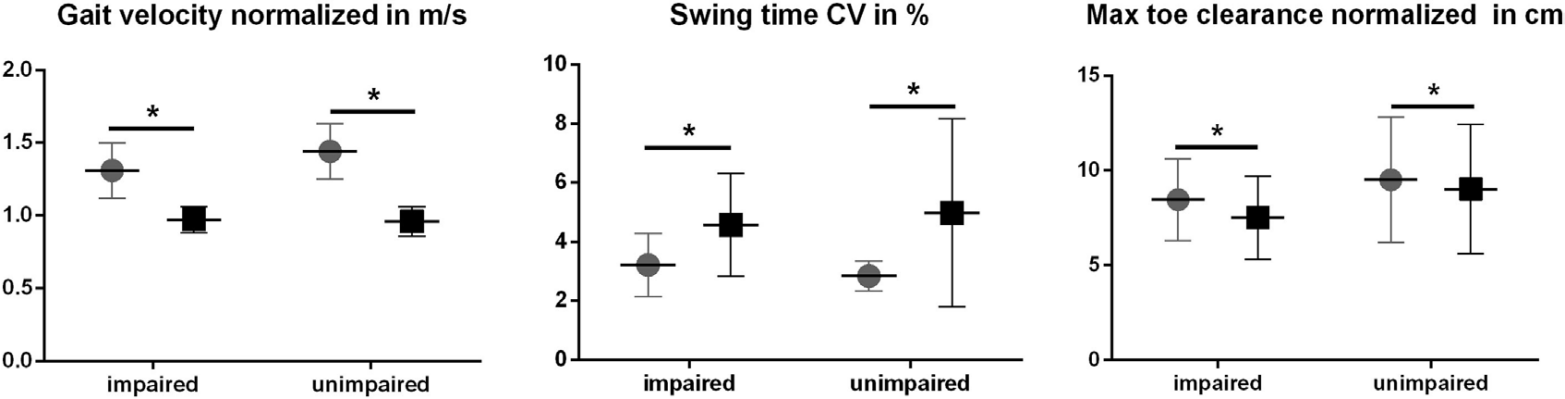
Selected spatiotemporal gait parameters in single 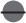 and dual task ◼ condition (cognitively impaired, *n* = 21, and unimpaired Parkinson’s disease patients, *n* = 21). *Adapted significance level (α_C_) by Benjamini–Hochberg multiple testing correction (see Table 3).

Dual task costs for each gait parameter were used to evaluate the effect of the DT in cognitively impaired and unimpaired PD patients. One-way ANOVA and Mann–Whitney *U*-test did not reveal significant between-group effects in DT costs of the gait parameters analyzed (Table 5). Interestingly, DT costs for stride time, gait velocity, and all gait variability parameters were larger in the cognitively unimpaired group. In DT condition, gait velocity significantly reduced in both groups, by 24% in the cognitively impaired and by 32% in the unimpaired group. Swing time CV substantially increased by 57% in cognitively impaired and by 74% in cognitively unimpaired PD patients. In contrast, the decrease of max TC was larger in cognitively impaired patients (12%) compared with the unimpaired group (5%). Differences in gait velocity, swing time variability, and max TC between groups and conditions are shown in Figure 3.

**Table 5:** Dual task costs (in %) in cognitively impaired (MoCA < 26, *n* = 21) and unimpaired (MoCA ≥ 26, *n* = 21) Parkinson’s disease patients. Means were compared using one way ANOVA and Mann–Whitney *U*-test.

Gait parameter	MoCA < 26		MoCA ≥ 26		*F* (1,40); *Z* value^a^	*p*	η^2^	α_C_
	Mean (%)	SD	Mean (%)	SD				
Stride time normalized	27.3	24.8	40.4	25.4	2.845	0.099	0.066	0.012
Stride time CV	56.6	75.0	93.7	116.0	−1.031^a^	0.302	0.025	0.027
Swing time	−2.4	1.8	−2.9	3.2	−1.069^a^	0.285	0.027	0.023
Swing time CV	56.6	78.0	74.1	103.1	−0.075^a^	0.940	0.000	0.050
Stance time	1.4	1.0	1.6	1.7	−1.270^a^	0.204	0.038	0.019
Stance time CV	52.5	87.6	84.0	107.9	−0.641^a^	0.521	0.010	0.038
Stride length normalized	−7.5	5.3	−7.1	6.3	0.036	0.851	0.001	0.046
Stride length CV	11.6	49.9	30.6	96.2	−0.767^a^	0.443	0.014	0.035
HS angle	−13.0	14.5	−1.4	29.5	2.601	0.115	0.061	0.015
TO angle	−5.5	3.8	−6.4	2.6	0.742	0.394	0.018	0.031
max TC normalized	−11.9	8.8	−5.2	12.2	4.270	0.045	0.096	0.004
Cadence normalized	−8.4	5.6	−9.5	6.9	−0.340^a^	0.734	0.003	0.042
Gait velocity normalized	−24.1	15.0	−32.2	11.2	3.762	0.060	0.086	0.008

*HS, heel strike; TO, toe off; max TC, maximum toe clearance; CV, coefficient of variance; SD, standard deviation*.

*Significance level was adapted by Benjamini–Hochberg correction for multiple comparisons (α_C_). Bold numbers indicate significance*.

*^a^Mann–Whitney U-test*.

## Discussion

This study investigated the interplay of cognitive deficits in PD and gait performance during a widely used DT paradigm. We hypothesized that PD patients with cognitive deficits show larger DT costs in gait parameters. However, our results did not reveal correlations between DT costs of gait parameters and cognitive performance assessed with MoCA. DT gait costs combined with clinical confounders explained about 30% of the MoCA score variance. Comparing matched groups of cognitively impaired and unimpaired PD patients, we did not find significant differences in DT gait costs. Confounder effects on DT walking as previously described (17, 24) were excluded since we matched groups by clinical confounders such as age, gender, disease duration, H&Y, UPDRS-III, medication and depressive symptoms. These findings suggest that DT gait performance is not indicative for cognitive impairment in PD assessed with MoCA. There are several considerations to be drawn based on the present finding.

Cognitive performance of PD patients was particularly affected in the MoCA subdomains visuospatial/executive function, attention, language, abstraction, and memory. The performance in these subdomains is consistent with a larger PD cohort (*n* = 486) (44). Interestingly, in our study cognitive impairment is inadequately reflected by DT gait costs. The cognitively impaired group reached a significant lower score in the MoCA task “serial 7 subtraction” compared with the unimpaired group. It has been shown that serial subtraction in steps of 3 and steps of 7 while walking has a substantial effect on gait in PD (6). Since serial 7 subtraction is more challenging than the DT “serial 3 subtraction,” the comparability of the cognitive performance between MoCA task and the cognitive task of the DT is limited. However, even the less challenging serial 3 subtraction substantially distracted the patients and aggravated the gait impairment (large effect sizes for DT on gait parameters). Intriguingly, this effect was present in both groups, e.g., independently from the cognitive performance assessed by MoCA. This suggests that motor-cognitive DT during walking may not be sensitive enough to detect mild cognitive impairment in PD patients. Additional investigations are needed to explore the role of different challenging motor-cognitive DTs to address their sensitivity to detect mild cognitive impairment in PD.

A direct relationship between cognitive deficits and gait impairment has been described in patients with dementia (12). Attention and executive function deficits are associated with basal ganglia pathology (14). Both cognitive domains are impaired in PD (45) and necessary to appropriately allocate cognitive resources for gait control in single and DTs (15, 46). We used the MoCA score in this study since this test adequately detects mild cognitive impairment in PD (35). Significant differences in DT gait costs could not be detected although matched groups significantly differed in the MoCA subsections “executive function” and “attention.” However, limits of the MoCA in detecting executive function and attention that are relevant for motor control should be taken into account. Impaired executive functions assessed by a trail making test have been shown to negatively influence DT gait costs in PD (6). In the MoCA, a short version of trail making is also included but maybe executive functions necessary for motor control during DT may not be sufficiently detected with this version. The MoCA as global cognitive assessment used in the clinical routine may not be as precise as extensive neuropsychological test batteries (47, 48). In a recent study using a neuropsychological test battery including trail making for executive function, verbal episodic memory, naming, attention, and working memory tests, DT gait has been shown to correlate with progression in dementia in older adults (*n* = 112) with mild cognitive impairment (49). This indicates on the one hand that more precise assessments might be necessary to detect cognitive deficits specific for DT gait. On the other hand, patients with progressed dementia were particularly impaired in naming and attention but not in executive functions indicating that other parts of cognitive-motor control are affected compared with PD patients. In general, it should be taken into account that the MoCA may be a global cognitive assessment tool but not sensitive enough to detect executive functions responsible for DT gait performance in PD.

PD patients were not instructed to prioritize walking or counting during the DT. In this study, DT costs for gait parameters were recorded, but DT costs for the cognitive task (incorrect answers in counting backwards) were not covered. Therefore, it may be possible that PD patients differently prioritized cognitive and motor task. Bloem et al. (50, 51) reported that PD patients may use the so called “posture second strategy,” where they focus less on maintaining a safe gait compared with healthy controls. We observed similar gait performance in both groups during DT walking and found descriptively larger DT costs of gait velocity in the cognitively unimpaired group which may indicate that this group focused more on the cognitive task. In this context, it has also been described that motor-cognitive DTs show twofold effects on gait performance. They reflect a PD-independent, age-related reduction in gait parameters, and a PD-specific coordination deficit during DT that affects postural control (52). Smulders et al. (53) observed that DT performance in the gait or cognitive task does not correlate with prospective falls in a large PD cohort (*n* = 263). This contradicts previous studies which observed associations between fall risk and DT gait (9–11). Taken together, in the light of these studies our results suggest that DT gait performance should be carefully interpreted in the clinical context due to variable individual responses under this challenge. DTs have the potential to reveal motor-cognitive deficits that are linked to basal ganglia pathology; however, appropriate assessments of gait and cognitive performance are necessary, and influencing factors should be controlled. Future studies should strictly control task prioritization by recording DT costs for gait and cognition.

Correlation analysis did not reveal significant correlations between MoCA score and DT gait costs. However, DT costs of stride length, gait velocity, swing time CV, and max TC showed at least weak correlations to cognitive impairment in this cohort and have previously been described as parameters that are affected by DT walking in PD (6–8). Future studies should focus on DT costs of those gait parameters and evaluate the predictive value in terms of cognitive impairment. Max TC may be an interesting parameter with regard to fall risk in PD during DT. Trip risk and falls in cognitively impaired PD patients are not well understood due to the fact that PD patients with cognitive impairment are often excluded in fall-related studies in PD (54). Further research should focus on the relevance of foot clearance to detect trip risk during DT walking in cognitively impaired PD patients.

## Conclusion

The results of this study indicate that DT gait performance did not reflect cognitive impairment in PD assessed by MoCA. Instead, cognitively impaired and unimpaired PD patients were equally challenged by the DT. Differences in task prioritization and less sensitive cognitive assessment using MoCA to evaluate executive functions specific for DT gait control may have influenced the results. Furthermore, DT gait parameters should be carefully interpreted in the clinical context due to variable individual responses to DTs.

## Ethics Statement

The study was approved by the local ethics committee (IRB-approval-Re. No. 4208, IRB, Medical Faculty, Friedrich-Alexander University Erlangen-Nürnberg, Germany), and all participants gave written informed consent according to the Declaration of Helsinki.

## Author Contributions

HG was responsible for conception, design, and organization of the study. He performed data acquisition, statistical analysis, and interpretation, and wrote the manuscript. FM, JS, and ZK supported data acquisition and reviewed the manuscript. BE, WA, KP, and SS collaborated in this project, critically discussed findings, and reviewed the manuscript from a technical, statistical, and movement science perspective. JW and JK supported in designing the study, critical revision, and preparing the manuscript.

## Conflict of Interest Statement

HG, KP, JS, ZK, WA, and SS declare no conflict of interest. FM is supported by the Interdisciplinary Center For Clinical Research (IZKF) of the University Hospital Erlangen. BE holds ownerships of Portabiles HealthCare Technologies GmbH and Portabiles GmbH, received compensation and honoraria from serving on scientific advisory boards for Abbvie GmbH, adidas GmbH, Bosch Sensortec GmbH, and ST Sportservice GmbH. JW reports personal fees outside of the submitted work from Teva GmbH, Ever Pharma GmbH, Desitin Arzneimittel GmbH, Abbvie GmbH & Co. KG, Biogen GmbH, and GlaxoSmithKline GmbH & Co. KG. JK holds ownerships of Portabiles HealthCare Technologies GmbH and Portabiles GmbH, received compensation and honoraria from serving on scientific advisory boards for LicherMT GmbH, Abbvie GmbH, UCB Pharma GmbH, GlaxoSmithKline GmbH & Co. KG, Athenion GmbH, and Thomashilfen GmbH; as well as lecturing from UCB Pharma GmbH, TEVA Pharma GmbH, Licher MT GmbH, Desitin GmbH, Abbvie GmbH, Solvay Pharmaceuticals, and Ever Neuro Pharma GmbH.

## Abbreviations

PD, Parkinson’s disease; ST, single task; DT, dual task; UPDRS-III, Unified Parkinson Disease Rating Scale part III (motor score); H&Y, Hoehn and Yahr disease stage; MoCA, Montreal Cognitive Assessment; SDS, Zung Depression scale; LEDD, levodopa equivalent daily dose; η^2^, effect size eta squared; α_C_, adapted significance level by Benjamini–Hochberg multiple testing correction.
